# The Use of Person-Centered Language in Medical Research Journals Focusing on Psoriasis: Cross-sectional Analysis

**DOI:** 10.2196/28415

**Published:** 2021-06-11

**Authors:** Ryan Ottwell, Benjamin Heigle, Arjun K Reddy, Nicholas Sajjadi, Alexis Wirtz, Courtney Cook, Hannah Howard, Micah Hartwell, Matt Vassar

**Affiliations:** 1 Oklahoma State University Center for Health Sciences Tulsa, OK United States

**Keywords:** psoriasis, dermatology, person-centered language, stigma, inclusive language, language

## Abstract

**Background:**

Person-centered language places a person’s identity before any disability or medical condition they may have. Using person-centered language reduces stigma and improves the patient-physician relationship, potentially optimizing health outcomes. Patients with psoriasis often feel stigmatized due to their chronic skin condition.

**Objective:**

We seek to evaluate the use of person-centered language in psoriasis literature and to explore whether certain article characteristics were associated with non–person-centered language.

**Methods:**

We performed a systematic search on PubMed for recently published articles in journals that regularly publish psoriasis studies. After article reduction procedures, randomization, and screening, we reached our target sample of 400 articles. The following non–person-centered language terms were extracted from each article: “Psoriasis Patient,” “Psoriasis subject,” “Affected with,” “Sufferer,” “Suffering from,” “Burdened with,” “Afflicted with,” and “Problems with.” Screening and data extraction occurred in a masked duplicate fashion.

**Results:**

Of the 400 included articles, 272 (68%) were not adherent to person-centered language guidelines according to the American Medical Association Manual of Style. The most frequent non–person-centered language term was “Psoriasis Patient,” found in 174 (43.5%) articles. The stigmatizing language was associated with the type of article and funding status, with original investigations and funded studies having higher rates of stigmatizing language.

**Conclusions:**

Articles about psoriasis commonly use non–person-centered language terms. It is important to shift away from using stigmatizing language about patients with psoriasis to avoid potential untoward influences. We recommend using “patients with psoriasis” or “patient living with psoriasis” to emphasize the importance of person-centered care.

## Introduction

It is estimated that 125 million people worldwide have psoriasis [1]—a chronic skin condition associated with arthritic disease; cardiovascular disease; and, namely, psychiatric disorders like depression [2]. Indeed, depression occurs in 9% to 55% of patients with psoriasis, and the impact of having psoriasis on the overall quality of life is comparable to that of patients with cancer [2-5]. Additionally, psychiatric morbidity in patients with chronic skin diseases, like psoriasis, is significantly associated with poorer medical compliance [6], which may lead to poorer health outcomes. The psychiatric distress experienced by patients with psoriasis may be exacerbated by feelings of stigma associated with psoriasis [7]. In numerous studies, patients living with psoriasis have reported feeling stigmatized due to this chronic skin condition [3-5]. Thus, reducing stigma among patients with psoriasis may serve to minimize untoward psychosocial influences and to optimize health outcomes.

Stigma is defined as “a mark of disgrace associated with a particular circumstance, quality, or person” [8], and the application of a generalized stigma to medical conditions may lead to decreased patient self-esteem, support, and likelihood of seeking medical care. Oftentimes, stigmatizing language is perpetuated by its widespread use in medical literature, which flows into medical education and ultimately into patient interactions [7]. The use of stigmatizing language is known to occur in other medical fields and is associated with negative health outcomes [3,6]. To decrease stigma experienced by patients with psoriasis in the dermatologic community, it is imperative to limit the use of stereotyping labels and to instead place an emphasis on the use of person-first language or person-centered language.

Person-centered language is based on the notion that it is most appropriate to place individuals ahead of the disabilities or medical conditions they have [9]. To treat individuals with psoriasis appropriately, we must first recognize the proper way to refer to them [10]. In 2010, the American Psychological Association defined the use of person-centered language, stating that the goal is to “maintain integrity of the individuals as human beings and to avoid language that objectifies a person by his or her condition” [11]. Similarly, many scholarly journals have begun to require the use of person-centered language in manuscripts submitted for publication [12], and the American Medical Association Manual of Style (AMAMS) requires authors to follow guidelines that include the avoidance of labeling people with their disabilities or diseases [13]. The use of person-centered language regarding patients with psoriasis is essential in fostering an advantageous relationship between the practitioner and patient. Most importantly, using person-centered language can promote a favorable environment for improving the overall well-being and quality of life for patients treated for psoriasis.

Thus, the primary objective of our study is to explore the use of person-centered language in journals that have published the most articles on psoriasis over the past 2 years. Additionally, we examined whether associations between person-centered language and particular study characteristics exist. Identifying areas for improvement regarding the use of person-centered language in the dermatologic community is necessary to reduce stigma experienced by patients with psoriasis.

## Methods

Using a cross-sectional design, one author (MH) conducted a systematic search via PubMed on May 7, 2020. To include a broad range in the initial query, we searched for the term “psoriasis” in the title or abstract of articles with filters to include studies of humans that were available in English from May 1, 2018, to April 30, 2020. For article reduction, we included journals with 20 or more search returns to capture studies from journals who regularly publish psoriasis-related articles. The remaining articles were then randomized and distributed to authors (AR and BH) separately for article screening and data extraction, which were conducted in a masked duplicative fashion. Articles were screened until a final sample size of 400 articles was achieved among both authors, who then met for reconciliation of responses. For an article to be included the following criteria must be met: the article pertains to the topic of psoriasis, the article involves human subjects, and the article is available in English. All peer-reviewed original research articles, including research letters, brief reports, case reports, published abstracts, and commentaries pertaining to psoriasis were included. Extracted information included the article type, study method, type of intervention, funding source, country of the first author, and whether the article mentioned adherence to reporting guidelines.

To analyze person-centered language among articles, we systematically searched each article for a list of non–person-centered language labels and stigmatizing and euphemistic language that were established a priori to the data process. Searched terms were “Psoriatic(s),” “Psoriasis Patient,” “Psoriasis subject,” “Affected,” “Sufferer,” “Suffering from,” “Burdened with,” “Afflicted with,” and “Problems with.” Regarding the search term “Psoriatic,” this includes referring to patients as either “psoriatics” or as a “psoriatic patient.” Following completion of data extraction, investigators were unmasked and data reconciliation occurred to resolve any disagreements between investigators. If an agreement could not be reached, a third-party arbitrator (author RO) was consulted for adjudication.

Following data extraction, we calculated the proportion of articles with and without deviation from the AMAMS [13] guidelines compared to the total number of articles in this sample. Additionally, we evaluated the most common forms of non–person-centered language terminology used among these articles. Next, we measured the associations between adherence to person-centered language guidelines and extracted study characteristics using chi-square tests. The journal reduction process, article randomization, and statistical analyses were performed using STATA 16.1 (StataCorp) on February 19, 2021.

## Results

**Figure 1:**
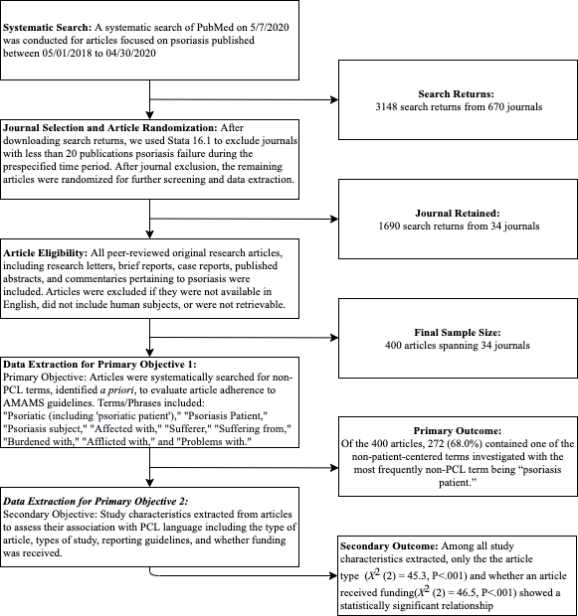
Flowchart of systematic investigation and outcomes of PCL in publications focused on psoriasis according to the AMAMS. AMAMS: American Medical Association Manual of Style; PCL: person-centered language.

Our query resulted in 3148 search returns from 670 journals. After article reduction procedures, randomization, and screening, we reached our target sample of 400 articles, which spanned 34 journals (Figure 1). A majority of the articles were original research (n=270, 67.5%; Table 1).

**Table 1:** Characteristics of studies and frequency of adherence to PCL.

Characteristics		Articles (N=400), n (%)	Articles with PCL^a^ adherence, n (%)	Articles that were non-PCL adherent, n (%)	Chi-square (*df*)		*P* value	
**Type of article**						45.3 (2)		<.001
	Case report	40 (10.0)	22 (5.5)	18 (4.5)				
	Editorial	90 (22.5)	49 (12.3)	41 (10.3)				
	Original research	270 (67.5)	57 (14.3)	213 (53.3)				
**Type of research**						46.5 (4)		<.001
	Clinical trial	49 (12.3)	12 (3)	37 (9.3)				
	Literature review	36 (9)	6 (1.5)	30 (7.5)				
	Editorials	131 (32.8)	71 (17.8)	60 (15)				
	Observational	156 (39)	30 (7.5)	126 (31.5)				
	Systematic review or meta-analysis	28 (7)	9 (2.3)	19 (4.8)				
**Type of intervention**						2.5 (3)		.48
	Drug/pharmacologic	132 (33)	48 (12)	84 (21)				
	Multiple therapies	7 (1.8)	2 (0.5)	5 (1.3)				
	No treatment	250 (62.5)	76 (19)	174 (43.5)				
	Nonpharmacologic	11 (2.9)	2 (0.6)	9 (2.3)				
**Adherence to reporting guidelines**						0.00 (1)		.95
	Not mentioned	384 (96)	123 (30.8)	261 (65.3)				
	Yes	16 (4)	5 (1.3)	11 (2.8)				
**Study was funded**						11.5 (1)		.001
	No	239 (59.8)	92 (23)	147 (36.8)				
	Yes	161 (40.3)	36 (9)	125 (31.3)				

^a^PCL: person-centered language.

The most prevalent type of research was cross-sectional or observational (n=156, 39%) followed by editorials (n=131, 32.8%). Of the 400 articles, 250 (62.5%) were not interventional studies, 384 (96%) did not mention adherence to any reporting guidelines, and 239 (59.8%) were not funded. According to the first author’s affiliation, the majority of the articles were from the United States, Japan, and Italy (Table 2).

**Table 2:** Use of PCL by country.

Country^a^	Articles (N=400), n	Articles with PCL^b^ adherence, n (%)	Articles that were non-PCL adherent, n (%)
Australia	5	3 (60)	2 (40)
Austria	3	2 (67)	1 (33)
Belgium	3	1 (33)	2 (67)
Brazil	5	0 (0)	5 (100)
Canada	22	8 (36)	14 (64)
Chile	1	0 (0)	1 (100)
China	23	6 (26)	17 (74)
Czech Republic	1	0 (0)	1 (100)
Denmark	22	5 (23)	17 (77)
Egypt	13	4 (31)	9 (69)
Estonia	1	0 (0)	1 (100)
France	12	4 (33)	8 (67)
Germany	23	4 (17)	19 (83)
Greece	3	2 (67)	1 (33)
Hungary	2	1 (50)	1 (50)
India	5	3 (60)	2 (40)
Ireland	11	7 (64)	4 (36)
Israel	2	0 (0)	2 (100)
Italy	28	9 (32)	19 (68)
Japan	31	12 (39)	19 (61)
Netherlands	3	1 (33)	2 (67)
New Zealand	2	0 (0)	2 (100)
Norway	6	1 (17)	5 (83)
Pakistan	1	0 (0)	1 (100)
Poland	7	0 (0)	7 (100)
Portugal	2	0 (0)	2 (100)
Scotland	1	0 (0)	1 (100)
Singapore	1	0 (0)	1 (100)
Slovenia	1	0 (0)	1 (100)
South Africa	1	0 (0)	1 (100)
South Korea	10	4 (40)	6 (60)
Spain	24	11 (46)	13 (54)
Sweden	4	0 (0)	4 (100)
Switzerland	7	1 (14)	6 (86)
Taiwan	9	1 (11)	8 (89)
Thailand	3	1 (33)	2 (67)
Turkey	7	1 (14)	6 (86)
UK	18	7 (39)	11 (61)
Ukraine	1	0 (0)	1 (100)
US	75	28 (37)	47 (63)
Venezuela	1	1 (100)	0 (0)
Total	400	128 (32)	272 (68)

^a^Country determined by first author’s affiliation.

^b^PCL: person-centered language.

Of the 400 articles, 272 (68%) were not adherent to person-centered language guidelines according to AMAMS. Of these 272 articles with non–person-centered language, 129 (47.4%) included more than one non–person-centered language term. The most frequent non–person-centered language term identified was “Psoriasis Patient,” found in 174 (43.5%) of the 400 articles, followed by “Psoriatic(s),” which was found in 103 (25.75%) articles (Table 3).

**Table 3:** Non-PCL terms and frequency within psoriasis articles.

Non-PCL^a^ term searched	Articles in which non-PCL terms were present (N=400), n (%)
Psoriatic	103 (25.8)
Psoriasis patient	174 (43.5)
Psoriasis subject	2 (0.5)
Affected	63 (15.8)
Sufferer	2 (0.5)
Suffering from	39 (9.8)
Burden with	55 (13.8)
Afflicted with	2 (0.5)
Problem with	0 (0.0)

^a^PCL: person-centered language.

Significant associations were found between adherence to person-centered language guidelines and the type of article (χ^2^_2_=45.3; *P*<.001), as original research showed a higher proportion of studies with non–person-centered language terminology, and between person-centered language and type of research (χ^2^_4_=46.5; *P<*.001), where observational studies also contained a larger proportion of non–person-centered language studies. Additionally, there was a significant relationship between person-centered language and an article being funded (χ^2^_1_=11.5; *P*=.001) in which 38.5% (92/239) of the nonfunded articles were person-centered language adherent, compared to 22.4% (36/161) of the studies that were funded.

## Discussion

We found that over two-thirds of the articles in our study contained non–person-centered language when referring to patients living with psoriasis. The most common non–person-centered language labels were “psoriasis patient” and “psoriatic”—to include “psoriatic patient” or “psoriatic subject.” Efforts are needed to reduce the use of stigmatizing language in the medical community to prevent perpetuating non–person-centered language in medical literature and medical education. Clinicians and researchers may benefit from understanding that terms such as “psoriatic” or “psoriasis patient” are potentially stigmatizing to patients with psoriasis. Understanding that these terms are prevalent and undesirable may promote changing how we refer to patients with psoriasis. Additionally, the inappropriate use of stigmatizing language by medical professionals in medical records may elicit the clinician bias, leading to lower quality care for patients [14].

To our knowledge, no study has explored the use of stigmatizing and euphemistic language in medical literature about psoriasis or its influence on patients with psoriasis. Although the influence of non–person-centered language on patients with psoriasis is unknown, using stigmatizing language is known to have negative impacts on patients with other disorders. For example, patients with substance abuse disorders being referred to as “addicts” is associated with reduced medical compliance, lower quality care by clinicians secondary to bias, and poorer overall health outcomes [14-18]. In a specific case, the prevalence of stigmatizing language in recent publications regarding alcohol use disorder remains high [19]. The use of non–person-centered language in recent publications emphasizes the need to intentionally use inclusive language in scientific literature. Ensuring the proper use of person-centered language in scientific literature may require journals to update author guidelines. Additionally, reviewers may need to be more vigilant for non–person-centered language terms and labels when reviewing articles.

To increase the use of person-centered language in the field of dermatology, we recommend that the ubiquitous use of “patients with psoriasis” or “patients living with psoriasis” replace terms like “psoriatic patient” or “psoriatic subject” when publishing medical literature. Advocating for widespread implementation of person-centered language–specific reporting guidelines for dermatology research is necessary for creating a person-centered, patient-first approach to caring for patients with psoriasis. By incorporating inclusive language in professional dialogue, person-centered language will trickle down into patient interactions, potentially leading to reduced stigma and increased positive outcomes for patients living with psoriasis. We believe it is important to emphasize that the use of non–person-centered language by health care professionals is likely not malicious and is mostly a remnant of an uninformed, unchanged status quo. As the culture of medicine continues to shift toward inclusive, patient-centered dynamics, it is increasingly important that the field of dermatology maintain a high standard of care by normalizing the use of inclusive, person-centered language.

This study is not without limitations. For example, the AMAMS’s definition of euphemistic language and emotional language is left to human interpretation and, therefore, subjective. Although we created a list of predefined non–person-centered language terms, other non–person-centered language terms may exist. An additional limitation lies within our study type; thus, our study’s results should not be generalized beyond what our findings suggest. Regarding our results, due to the nature of editorials, and the fewer expectations and parameters of nonfunded research, we expected a greater number of articles with non–person-centered language among them; however, original and funded research included more stigmatizing language. Based on our results, future research may be needed to investigate this phenomenon. Lastly, we only searched PubMed for our literature search. PubMed was chosen as it is one of the largest online research databases and has been used in previous person-centered language research [19]; however, some studies focused on psoriasis may have been excluded. Future studies focused on person-centered language in other dermatologic conditions and other medical fields in which the condition is subject to labeling stigma is warranted.

Patients with psoriasis often feel stigmatized due to their chronic skin condition. We searched the psoriasis literature base for a representative sample of the most recent psoriasis studies to evaluate the prevalence of stigmatizing, non–person-centered language. We found that the majority of articles in our sample contained some form of stigmatizing language regarding patients with psoriasis. Efforts are needed to shift from using stigmatizing language to using inclusive, person-centered language regarding people with psoriasis. Our findings may be useful to clinicians and researchers striving to provide patients with high-quality person-centered care by using language that is more inclusive and empathetic toward patients living with psoriasis.

## Abbreviations

AMAMS: American Medical Association Manual of Style
