# Correction: The Use of Person-Centered Language in Medical Research Journals Focusing on Psoriasis: Cross-sectional Analysis

**DOI:** 10.2196/31902

**Published:** 2021-07-16

**Authors:** Ryan Ottwell, Benjamin Heigle, Arjun K Reddy, Nicholas Sajjadi, Alexis Wirtz, Courtney Cook, Hannah Howard, Micah Hartwell, Matt Vassar

**Affiliations:** 1 Oklahoma State University Center for Health Sciences Tulsa, OK United States

In “The Use of Person-Centered Language in Medical Research Journals Focusing on Psoriasis: Cross-sectional Analysis” (JMIR Dermatol 2021;4(1):e28415) two errors were noted.

In the originally published manuscript, incorrect ORCID numbers were listed for authors Benjamin Heigle and Matt Vassar. The ORCID numbers have been corrected as follows:

Benjamin Heigle: 0000-0003-3724-0756

Matt Vassar: 0000-0003-2859-6152

The correction will appear in the online version of the paper on the JMIR Publications website on July 16, 2021, together with the publication of this correction notice. Because this was made after submission to PubMed, PubMed Central, and other full-text repositories, the corrected article has also been resubmitted to those repositories.

